# Performance Analysis and Four-Objective Optimization of an Irreversible Rectangular Cycle

**DOI:** 10.3390/e23091203

**Published:** 2021-09-12

**Authors:** Qirui Gong, Yanlin Ge, Lingen Chen, Shuangshaung Shi, Huijun Feng

**Affiliations:** 1 Institute of Thermal Science and Power Engineering, Wuhan Institute of Technology, Wuhan 430205, China; hbqi0913@163.com (Q.G.); geyali9@hotmail.com (Y.G.); shishuangshuang20@163.com (S.S.); huijunfeng@139.com (H.F.); 2 Hubei Provincial Engineering Technology Research Center of Green Chemical Equipment, Wuhan 430205, China; 3 School of Mechanical & Electrical Engineering, Wuhan Institute of Technology, Wuhan 430205, China

**Keywords:** finite time thermodynamics, rectangular cycle, power density, effective power, power output, thermal efficiency, multi-objective optimization

## Abstract

Based on the established model of the irreversible rectangular cycle in the previous literature, in this paper, finite time thermodynamics theory is applied to analyze the performance characteristics of an irreversible rectangular cycle by firstly taking power density and effective power as the objective functions. Then, four performance indicators of the cycle, that is, the thermal efficiency, dimensionless power output, dimensionless effective power, and dimensionless power density, are optimized with the cycle expansion ratio as the optimization variable by applying the nondominated sorting genetic algorithm II (NSGA-II) and considering four-objective, three-objective, and two-objective optimization combinations. Finally, optimal results are selected through three decision-making methods. The results show that although the efficiency of the irreversible rectangular cycle under the maximum power density point is less than that at the maximum power output point, the cycle under the maximum power density point can acquire a smaller size parameter. The efficiency at the maximum effective power point is always larger than that at the maximum power output point. When multi-objective optimization is performed on dimensionless power output, dimensionless effective power, and dimensionless power density, the deviation index obtained from the technique for order preference by similarity to an ideal solution (TOPSIS) decision-making method is the smallest value, which means the result is the best.

## 1. Introduction

After decades of development, a series of instructive and practical achievements have been obtained in finite time thermodynamics [1,2,3,4,5,6,7,8], the research objects of which include heat engines [9,10,11,12,13,14,15], refrigerators [16,17], heat pumps [18], chemical cycles [19], and quantum cycles [20,21]. The rectangular cycle (RC) is composed of four thermodynamic processes, its endothermic processes are closed to the endothermic processes of the dual cycle, and its exothermic processes are closed to the exothermic processes of the Miller cycle. Compared with the common internal combustion engine cycles, the cycle has no adiabatic process, so it is easier to realize in practical engineering. Since the cycle p−v diagram is rectangular, it is called the RC. Ferreira [22] first applied the classical thermodynamic theory to study the performance of the RC and derived the work output and thermal efficiency (TEF). Some scholars have introduced finite time thermodynamics theory into the performance analyses of the RC on this basis. Considering that the specific heats (SHs) of the working fluid (WF) were constants, Liu et al. [18] derived the work output and TEF of an endoreversible RC. Based on [23], Liu et al. [24] analyzed the influences of heat transfer loss and friction loss on the power output (POW) and TEF of an irreversible RC. Considering that the SH of WF were linear [25] and nonlinear [26] variable with temperature, Wang et al. [25,26] investigated the POW and TEF of endoreversible and irreversible RCs.

Yan [27] used P⋅η (where P is the cycle POW and η is the cycle TEF) as the objective function to study the performance of an endoreversible Carnot cycle in 1984, and Yilmaz [28] termed P⋅η as the effective power; then, the effective power was widely applied in the research of various heat engines [29,30,31,32,33].

In 1995, Sahin et al. [34] first put forward the power density (PD) (defined as the ratio of cycle POW to the maximum specific volume) as a performance indicator and compared the performance differences under the conditions of the maximum PD point and maximum POW point of the Joule–Brayton cycle, the results showing that optimizing the cycle with the goal of PD can reduce the size of the actual device, which plays a guiding role in the design of heat engines. Chen et al. [35] first introduced the PD into an internal combustion engine cycle and derived the TEFs of the reversible Atkinson cycle under the conditions of the maximum PD point and maximum POW point when any loss was not taken into account. Al-Sarkhi et al. [36] optimized the PD characteristics of a Miller cycle when any loss was not considered and obtained the TEF corresponding to the maximum PD point. Karakurt et al. [37] investigated the PD characteristics of a simple Brayton cycle when the WF was supercritical CO_2_. Shi et al. [38] derived the TEF corresponding to the maximum PD point of an irreversible Atkinson cycle considered when the SH was constant. Based on the RC model established in [23], Gong et al. [39] derived the relationships between the PD and expansion ratio, as well as between the PD and TEF, and compared the performance differences of the cycle at the maximum PD point and maximum POW point.

As the number of performance indicators of heat engines increases, it is necessary to obtain global optimization solutions of several objective functions when optimizing the performance of the heat engines. Compared with the NSGA, the improved multi-objective optimization (MOO) algorithm (NSGA-II) has a faster running speed and better solution set, so it is the first choice of the MOO algorithm [40,41,42,43,44,45,46,47,48,49,50,51,52,53,54,55,56,57,58,59,60,61]. Many scholars have applied NSGA-II to the performance optimizations of heat engines and then used several MOO decision-making methods to choose the optimal solution. Li et al. [49] established a regenerative Brayton cycle model which was driven by fossil fuels and solar energy and carried out MOO for the POW, TEF, and dimensionless thermal economic performance. Ahmadi et al. [50] applied NSGA-II to optimize the performance of the Atkinson cycle, carried out MOO for the cycle POW and TEF, and provided theoretical guiding significance for practical engineering. Based on the established irreversible Dual-Miller cycle model, Abedinnezhad et al. [51] carried out MOO for the TEF, ecological function, and ecological performance coefficient. Based on the established irreversible Atkinson cycle model with constant SH of WF, Shi et al. [38] applied NSGA-II to carry out MOO for the dimensionless POW, dimensionless PD, TEF, and dimensionless ecological function. Tang et al. [52] modeled the improved irreversible closed modified Brayton cycle when the heat source temperature was changed; derived the expressions of cycle dimensionless POW, dimensionless PD, TEF, and dimensionless ecological function; and then performed MOO on the cycle to obtain the optimal solutions of four-objective, three-objective, and two-objective optimizations. Yang et al. [53] considered five kinds of WF (refrigerant) that could be used for heat recovery of ORC (organic Rankine cycle), applied NSGA-II to carry out MOO for the LECTs (total savings of levelized electricity cost) and TEF of the cycle, and obtained the optimal working points under different refrigerants.

The above research shows that the effective power, PD, and MOO have been widely applied in performance analyses and optimizations of heat engines. However, research on effective power, PD, and MOO of the irreversible RC has not been reported in the published literature. RC is also known as a soft air-cycle heat engine [62], and it is useful for future soft robots due to their easy integration into soft structures and low-voltage power requirements. It is useful to further study the performance of the RC.

On the basis of the irreversible RC model with constant SH of WF built in [24], this paper will firstly obtain the analytical expressions of cycle PD and effective power by using finite time thermodynamics theory and compare the performance differences of the cycle under the conditions of the maximum PD, maximum effective power, and maximum POW; secondly carry out MOO for the dimensionless POW, TEF, dimensionless PD, and dimensionless effective power by taking the cycle expansion ratio as the optimization variable and applying the NSGA-II; and finally obtain the optimal solutions of four-objective, three-objective, and two-objective optimizations by using three decision-making methods.

## 2. Model and Performance Indicators of an Irreversible RC

**Figure 1:**
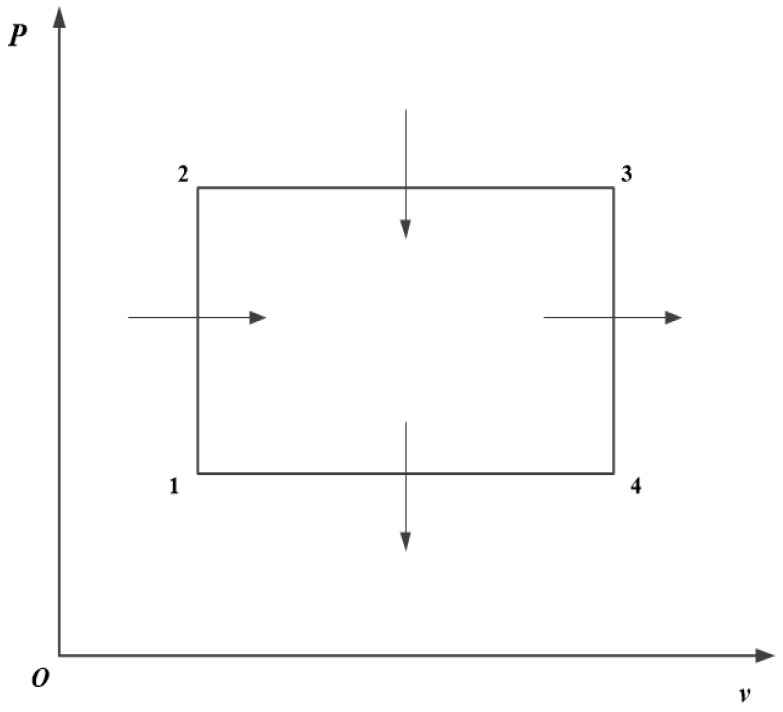
Cycle p−v diagram.

**Figure 2:**
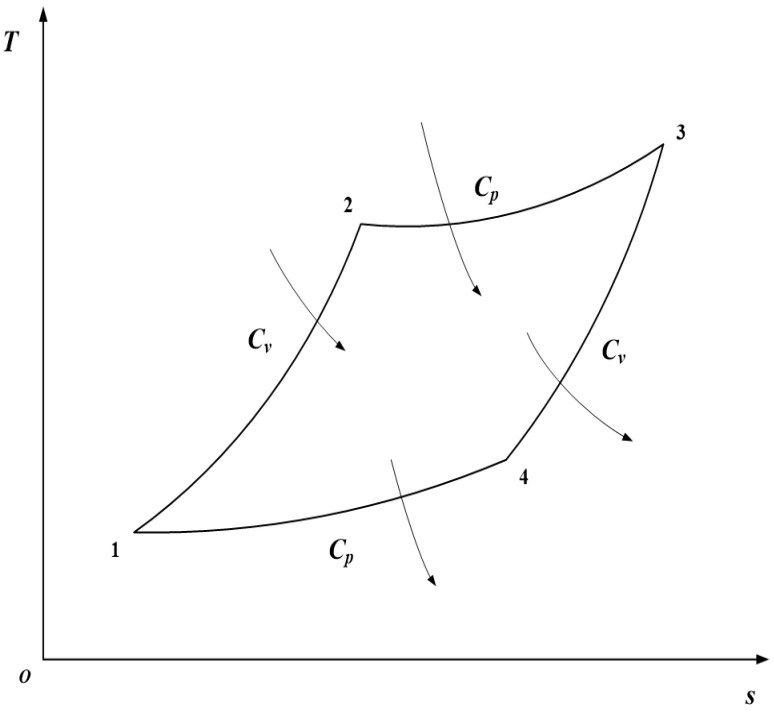
Cycle T−s diagram.

Figure 1 and Figure 2 show the irreversible RC model [24]. The cycle consists of two constant volume processes, 1→2 and 3→4, as well as two constant pressure processes, 2→3 and 4→1. Processes 1→2 and 2→3 are endothermic ones, and processes 3→4 and 4→1 are exothermic ones.

According to the state equation of ideal gas, one has

(1) $$v_{3}/v_{2}=T_{3}/T_{2}$$

(2) $$v_{4}/v_{1}=T_{4}/T_{1}=v_{3}/v_{2}$$

The expansion ratio of the RC is defined as $\gamma =v_{3}/v_{2}$, and the cycle temperature ratio (TR) is defined as $\tau =T_{3}/T_{1}$. The following equations can be obtained from Equations (1) and (2):

(3) $$T_{3}=\gamma T_{2}$$

(4) $$T_{4}=\gamma T_{1}$$

(5) $$T_{2}=\tau T_{1}/\gamma$$

The heat absorption rate of the WF is

(6) $${\dot{Q}}_{in}=\dot{m}\left[C_{v}(T_{2}-T_{1})+C_{p}(T_{3}-T_{2})\right]$$

The heat release rate of the WF is

(7) $${\dot{Q}}_{out}=\dot{m}\left[C_{v}(T_{3}-T_{4})+C_{p}(T_{4}-T_{1})\right]$$

where $\dot{m}$ is the mass flow rate of the WF, and $C_{p}$ and $C_{v}$ are the SHs at constant pressure and constant volume, respectively.

There is no loss in the ideal RC, but for the actual RC, it is necessary to consider the heat transfer loss between the WF and the external cylinder wall because the cylinder wall is not adiabatic, and the large temperature difference between the high-temperature WF and the outside makes this heat transfer loss not negligible. According to [63,64], it is assumed that the heat transfer loss through the cylinder wall is proportional to the temperature difference between the average temperature of the WF and the ambient temperature, and the heat transfer loss rate can be expressed as

(8) $${\dot{Q}}_{leak}=\left(T_{1}+T_{2}+T_{3}-3T_{0}\right)\frac{B}{3}=\left(T_{1}+T_{2}+T_{3}-3T_{0}\right)B_{1}$$

where $T_{0}$ is the ambient temperature, and $B=3B_{1}$ is the heat transfer loss coefficient between the external cylinder wall and the WF.

For the irreversible RC, it is also necessary to consider the friction loss between the piston and the cylinder wall. According to [65], the POW consumed by friction loss can be written as

(9) $$P_{\mu }=\frac{dW_{\mu }}{dt}=-\mu {\left(dx/dt\right)}^{2}=-\mu v^{2}$$

where μ is the friction loss coefficient.

The average piston speed can be expressed as

(10) $$\bar{v}=\frac{x_{1}-x_{2}}{\Delta t_{12}}=\frac{x_{2}(\gamma -1)}{\Delta t_{12}}$$

where $x_{1}$ and $x_{2}$ are the position of the piston at the maximum and minimum volume, and $\Delta t_{12}$ is the time consumed in the power stroke.

Substituting the average piston speed $\bar{v}$ with v in Equation (9), the cycle POW and TEF can be obtained as

(11) $$P={\dot{Q}}_{in}-{\dot{Q}}_{out}-P_{\mu }=\dot{m}(C_{p}-C_{v})(\gamma -1)(T_{2}-T_{1})-b{\left(\gamma -1\right)}^{2}$$

(12) $$\eta =\frac{P}{{\dot{Q}}_{in}+{\dot{Q}}_{leak}}=\frac{\dot{m}(C_{p}-C_{v})(\gamma -1)(T_{2}-T_{1})-b{\left(\gamma -1\right)}^{2}}{\dot{m}\left[(T_{3}-T_{2})C_{p}+(T_{2}-T_{1})C_{v}\right]+B_{1}(T_{1}+T_{2}+T_{3}-3T_{0})}$$

where $b=\mu {\left(x_{2}/\Delta t_{12}\right)}^{2}$.

According to the definition of PD in [34], the PD of the RC is

(13) $$P_{d}=\frac{P}{v_{4}}=\frac{P}{\gamma v_{1}}=\frac{\dot{m}(C_{p}-C_{v})(\gamma -1)(T_{2}-T_{1})-b{\left(\gamma -1\right)}^{2}}{\gamma v_{1}}$$

According to [35], the expressions of standardized dimensionless PD and dimensionless POW are written as

(14) $${\bar{P}}_{d}=P_{d}/{\left(P_{d}\right)}_{\max}$$

(15) $$\bar{P}=P/P_{\max}$$

According to the definition of effective power ($W_{ep}$) in [27,28], the $W_{ep}$ of the RC is

(16) $$W_{ep}=P\cdot \eta =\frac{{\left[\dot{m}(C_{p}-C_{v})(\gamma -1)(T_{2}-T_{1})-b{\left(\gamma -1\right)}^{2}\right]}^{2}}{\dot{m}\left[(T_{3}-T_{2})C_{p}+(T_{2}-T_{1})C_{v}\right]+B_{1}(T_{1}+T_{2}+T_{3}-3T_{0})}$$

According to [30], the expression of the dimensionless $W_{ep}$ is written as

(17) $${\bar{W}}_{ep}=W_{ep}/{\left(W_{ep}\right)}_{\max}$$

When γ, $T_{1}$, τ, and $T_{0}$ are given, the temperatures of each state point in the cycle can be calculated, and then the cycle POW, TEF, PD, and effective power can be obtained by substituting the calculation results into Equations (11)–(13) and (16).

## 3. Power Density and Effective Power Performance Analyses

According to [22,24,63,64,65], the parameters are as follows: $C_{p}=29.092\;J/(\mathrm{mol}\cdot K)$, $C_{v}=20.78\;J/(\mathrm{mol}\cdot K)$, γ=1.0~10.0, $T_{0}=300\;K$, $T_{1}=350\;K$, τ=4.2~6.2, b=32.5 W, B=2.2 W/K, and $\dot{m}=1\;\mathrm{mol}/s$.

### 3.1. Power Density Performance Analysis

**Figure 3:**
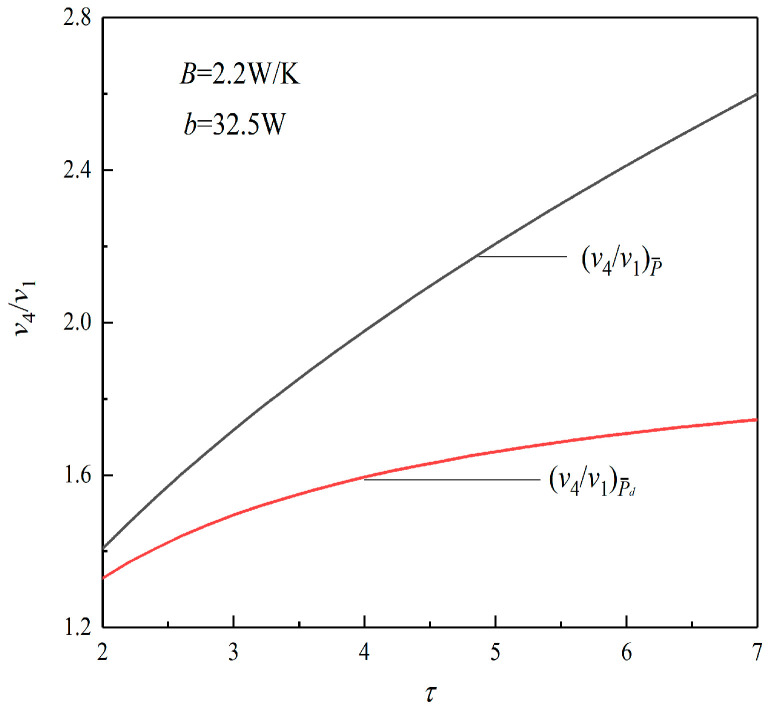
Relation between $v_{4}/v_{1}$ and τ.

Figure 3 shows the relationships between the maximum specific volume ratio ($v_{4}/v_{1}$) and τ under the conditions of ${\bar{P}}_{\max}$ and ${\left({\bar{P}}_{d}\right)}_{\max}$. It can be seen from the figure that, at the same TR (τ), the ${\left(v_{4}/v_{1}\right)}_{\bar{P}}$ corresponding to ${\bar{P}}_{\max}$ is always bigger than the ${\left(v_{4}/v_{1}\right)}_{{\bar{P}}_{d}}$ corresponding to ${\left({\bar{P}}_{d}\right)}_{\max}$. When τ is 6.2, ${\left(v_{4}/v_{1}\right)}_{\bar{P}}$ is 2.451, and ${\left(v_{4}/v_{1}\right)}_{{\bar{P}}_{d}}$ is 1.717, which decreases by about 29.95%, which means that the size of the heat engine is smaller when it works at the maximum PD point.

**Figure 4:**
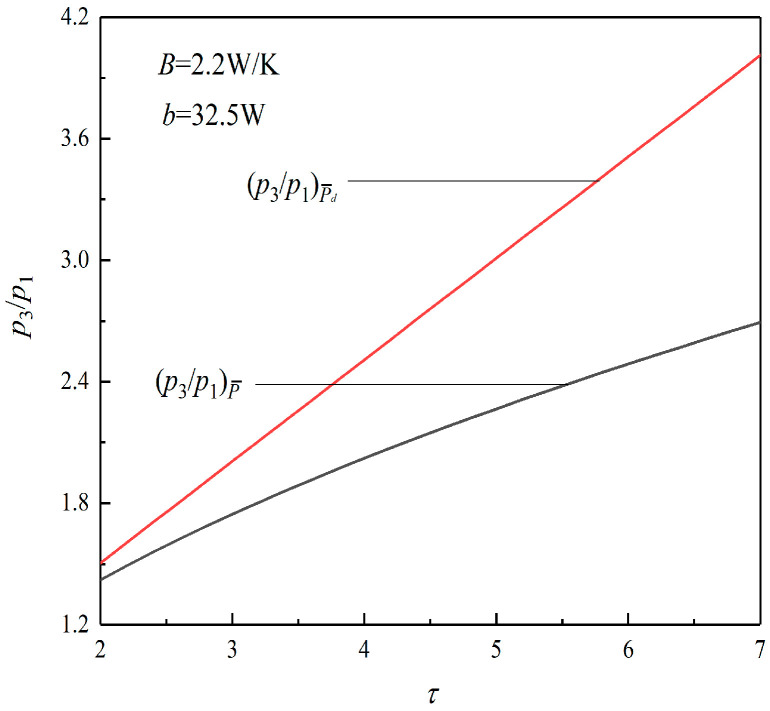
Relation between $p_{3}/p_{1}$ and τ.

Figure 4 shows the relationships between the maximum pressure ratio ($p_{3}/p_{1}$) and τ under the conditions of ${\bar{P}}_{\max}$ and ${\left({\bar{P}}_{d}\right)}_{\max}$. It can be seen from the figure that the ${\left(p_{3}/p_{1}\right)}_{\bar{P}}$ corresponding to ${\bar{P}}_{\max}$ is always less than the ${\left(p_{3}/p_{1}\right)}_{{\bar{P}}_{d}}$ corresponding to ${\left({\bar{P}}_{d}\right)}_{\max}$. When τ is 6.2, ${\left(p_{3}/p_{1}\right)}_{\bar{P}}$ is 2.53, and ${\left(p_{3}/p_{1}\right)}_{{\bar{P}}_{d}}$ is 3.61, which increases by about 42.69%, which means that although the heat engine has a smaller size under the condition of maximum PD, it is accompanied by a larger pressure ratio.

**Figure 5:**
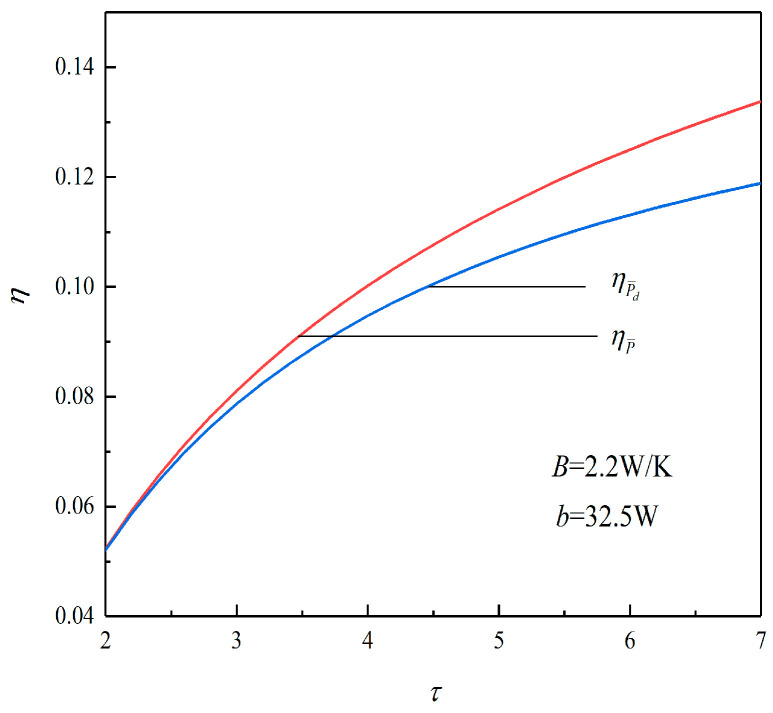
Relation between η and τ.

Figure 5 shows the relationships between the TEF versus τ under the conditions of ${\bar{P}}_{\max}$ and ${\left({\bar{P}}_{d}\right)}_{\max}$. It can be seen from the figure that the $\eta_{\bar{P}}$ corresponding to ${\bar{P}}_{\max}$ is always bigger than the $\eta_{{\bar{P}}_{d}}$ corresponding to ${\left({\bar{P}}_{d}\right)}_{\max}$. When τ is 6.2, $\eta_{\bar{P}}$ is 0.1269, and $\eta_{{\bar{P}}_{d}}$ is 0.1144, which decreases by about 9.8%.

According to Figure 3 and Figure 5, when τ is 6.2, $\eta_{{\bar{P}}_{d}}$ decreases by 9.8% compared with $\eta_{\bar{P}}$, and the TEF decreases slightly. However, ${\left(v_{4}/v_{1}\right)}_{{\bar{P}}_{d}}$ decreases by 28.95% compared with ${\left(v_{4}/v_{1}\right)}_{\bar{P}}$, and $v_{4}/v_{1}$ decreases greatly. This shows that when taking the maximum PD as the goal, although part of the TEF of the heat engine is sacrificed, the size of the heat engine decreases greatly.

### 3.2. Efficient Power Performance Analysis

**Figure 6:**
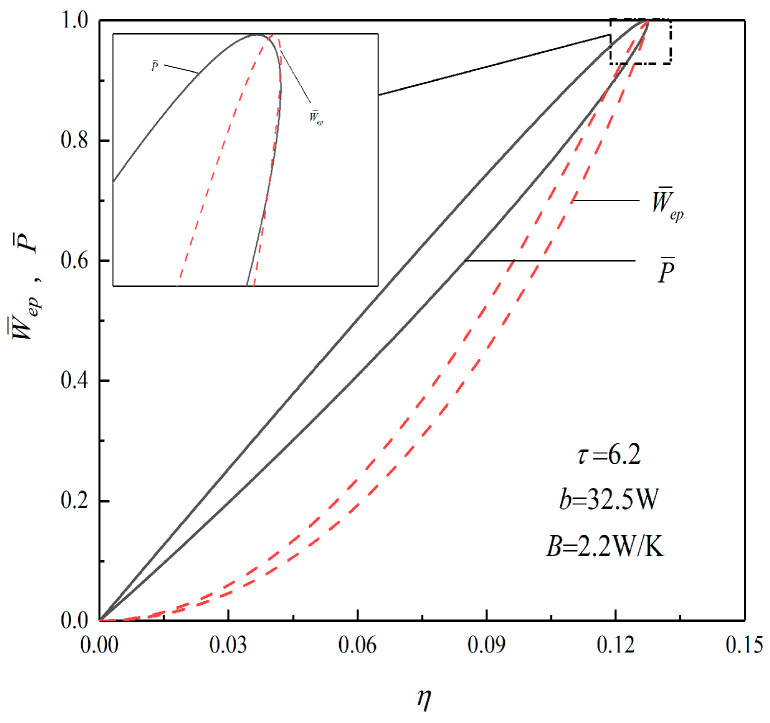
Relations of ${\bar{W}}_{ep}-\eta$ and $\bar{P}-\eta$ when τ=6.2.

Figure 6 shows the relationships of ${\bar{W}}_{ep}-\gamma$ and ${\bar{W}}_{ep}-\eta$ when τ=6.2. When τ=6.2, the dimensionless POW corresponding to the ${\left({\bar{W}}_{ep}\right)}_{\max}$ is 0.9990, the TEF corresponding to ${\left({\bar{W}}_{ep}\right)}_{\max}$ is 0.1273, and the TEF corresponding to ${\bar{P}}_{\max}$ is 0.1269. Compared with the maximum POW condition point, the POW corresponding to the ${\left({\bar{W}}_{ep}\right)}_{\max}$ decreases by 0.1%, and the TEF corresponding to the ${\left({\bar{W}}_{ep}\right)}_{\max}$ increases by 0.32%. Therefore, when the $W_{ep}$ is taken as the objective function, the cycle TEF can increase with sacrificing part of the POW, and the $W_{ep}$ reflects the compromise between the POW and TEF.

## 4. Multi-Objective Optimization

In this section, the γ is used as the optimization variable; the ${\bar{P}}_{d}$, $\bar{P}$, η, and ${\bar{W}}_{ep}$ are taken as the optimization goals; and the irreversible RC is optimized by using the “gamultiobj” algorithm that comes from the MATLAB software. Then, the corresponding Pareto frontiers are obtained, and the optimal solutions can be picked out by applying three decision-making methods of LINMAP, TOPSIS, and Shannon Entropy. Then, the three results obtained are compared.

Several MOO problems will arise when solving different combinations of optimization objectives.

Any combination of two objective functions can obtain six two-objective optimization expressions:

(18) $$\max\left\{ \begin{array}{l} \bar{P}(\gamma )\\ \eta (\gamma ) \end{array}\right.,\max\left\{ \begin{array}{l} \bar{P}(\gamma )\\ {\bar{P}}_{d}(\gamma ) \end{array}\right.,\max\left\{ \begin{array}{l} \bar{P}(\gamma )\\ {\bar{W}}_{ep}(\gamma ) \end{array}\right.,\max\left\{ \begin{array}{l} \eta (\gamma )\\ {\bar{P}}_{d}(\gamma ) \end{array}\right.,\max\left\{ \begin{array}{l} \eta (\gamma )\\ {\bar{W}}_{ep}(\gamma ) \end{array}\right.,\max\left\{ \begin{array}{l} {\bar{P}}_{d}(\gamma )\\ {\bar{W}}_{ep}(\gamma ) \end{array}\right.$$

Any combination of three objective functions can obtain four three-objective optimization expressions:

(19) $$\max\left\{ \begin{array}{l} \bar{P}(\gamma )\\ \eta (\gamma )\\ {\bar{P}}_{d}(\gamma ) \end{array}\right.,\max\left\{ \begin{array}{l} \bar{P}(\gamma )\\ \eta (\gamma )\\ {\bar{W}}_{ep}(\gamma ) \end{array}\right.,\max\left\{ \begin{array}{l} \bar{P}(\gamma )\\ {\bar{P}}_{d}(\gamma )\\ {\bar{W}}_{ep}(\gamma ) \end{array}\right.,\max\left\{ \begin{array}{l} \eta (\gamma )\\ {\bar{P}}_{d}(\gamma )\\ {\bar{W}}_{ep}(\gamma ) \end{array}\right.$$

Any combination of four objective functions can obtain one four-objective optimization expression:

(20) $$\max\left\{ \begin{array}{l} {\bar{P}}_{d}(\gamma )\\ {\bar{P}}_{d}(\gamma )\\ \eta (\gamma )\\ {\bar{W}}_{ep}(\gamma ) \end{array}\right.$$

**Figure 7:**
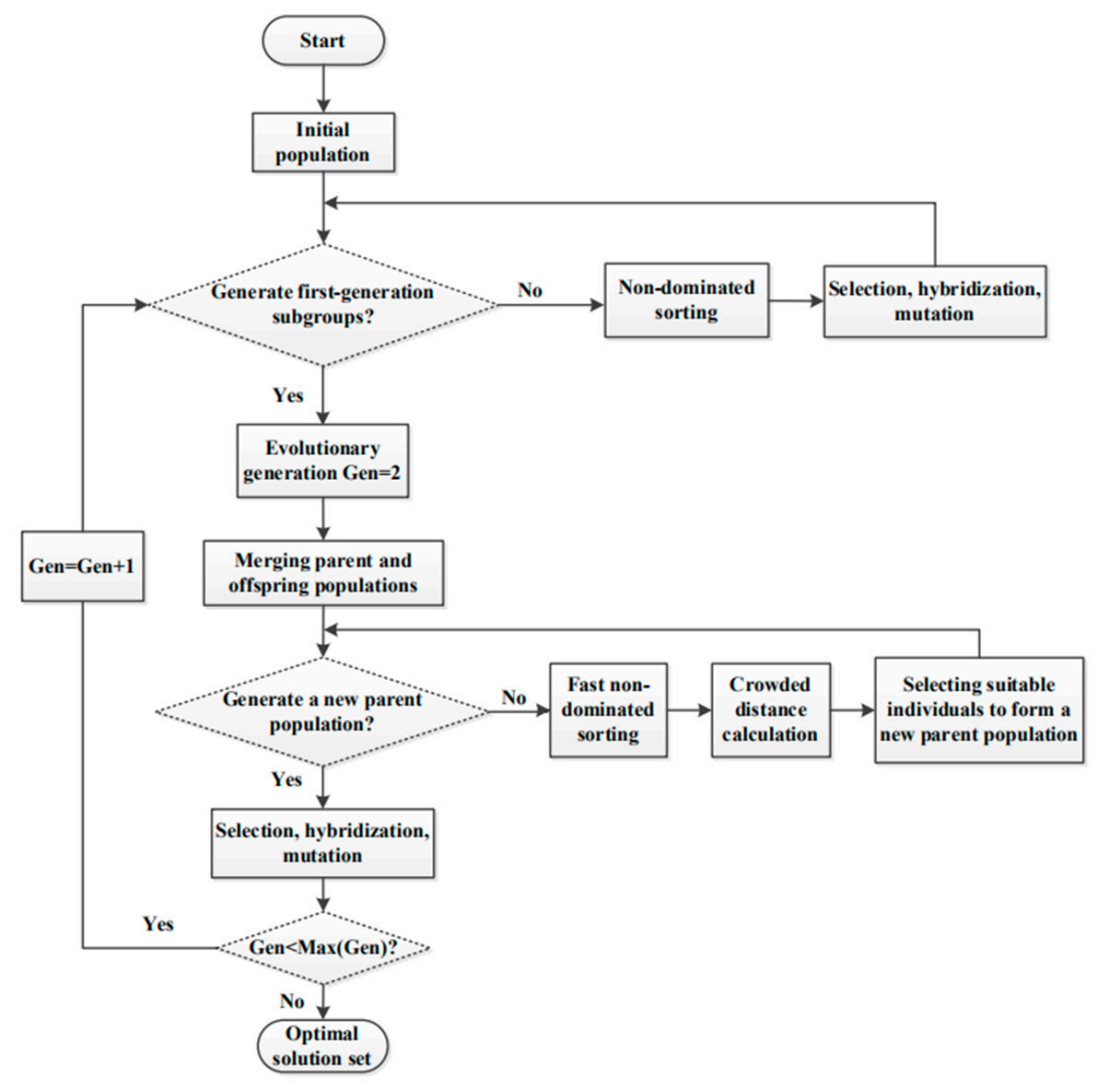
Flow chart of NSGA-II.

Figure 7 shows the complete process of NSGA-II [47]. Compared with the previous generation MOO evolutionary algorithm NSGA, the NSGA-II mainly makes the following three improvements:

(1) A new algorithm for fast non-dominant sorting is added, which greatly reduces the computational complexity.
(2) Elite strategy is introduced, and a new population is formed which is composed of two populations, the parent and the offspring populations, selecting superior individuals in the new population instead of selecting only in the offspring population, which not only expands the range of options but also reduces the selection loss of excellent individuals in the parent population
(3) Canceling the artificial designation of the shared parameters, which has been replaced by the congestion degree and the congestion degree comparison operator.

Table 1 lists the optimal solutions obtained by four-objective, three-objective, two-objective, and one-objective optimizations. The deviation index indicates the degree of deviation between the optimization result and the positive ideal point; the smaller the deviation index is, the closer the obtained optimization result is to the positive ideal point. Comparing the results of four-objective, three-objective, two-objective, and one-objective optimizations listed in Table 1, it can be found that when the MOO of $\bar{P}$, ${\bar{P}}_{d}$, and ${\bar{W}}_{ep}$ is performed, the deviation index under the TOPSIS method is the minimum, which means that its result is the best, and the MOO solution is better than the single-objective optimal solutions.

**Figure 8:**
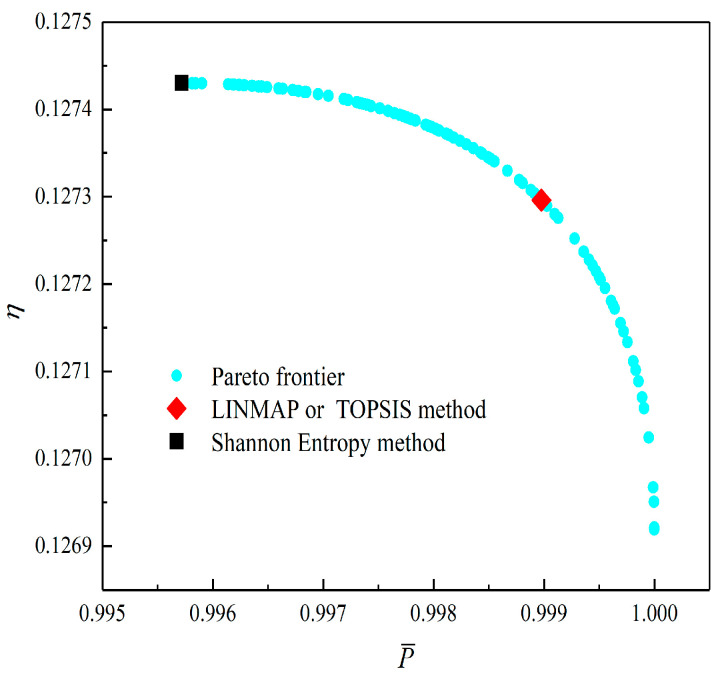
Two-objective optimization on $\bar{P}-\eta$.

**Figure 9:**
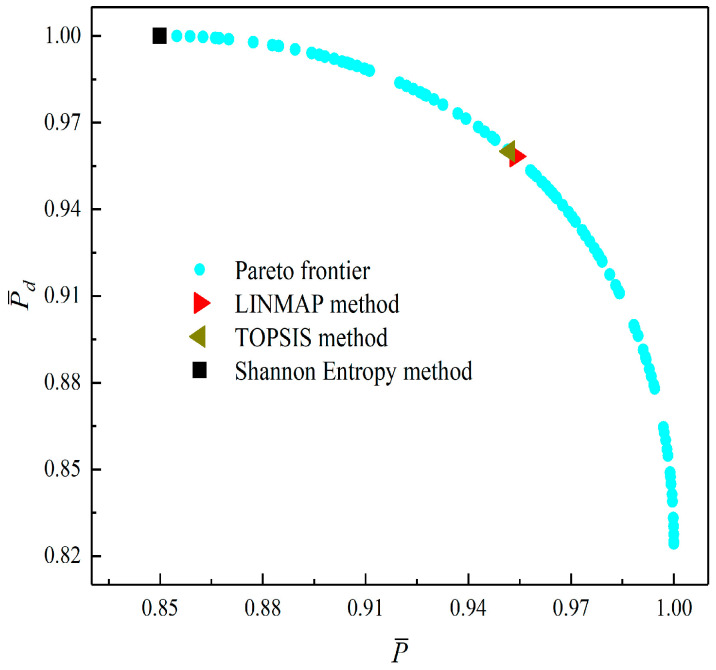
Two-objective optimization on $\bar{P}-{\bar{P}}_{d}$.

**Figure 10:**
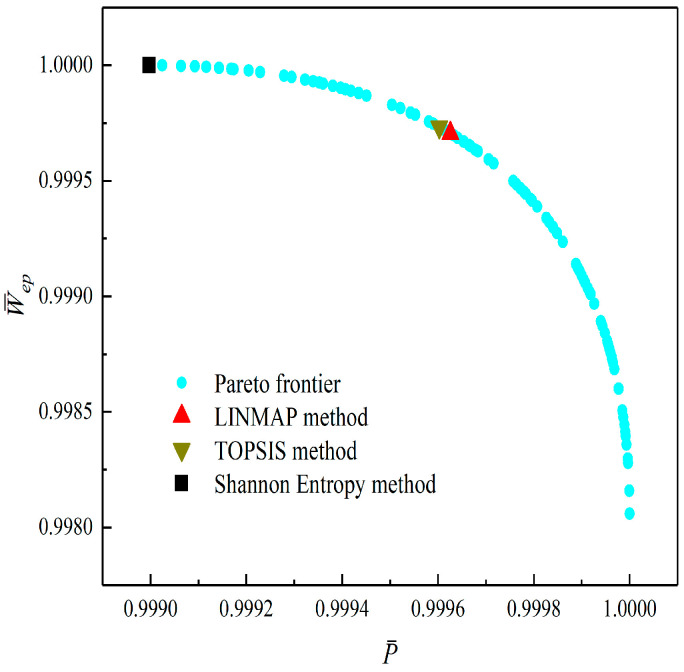
Two-objective optimization on $\bar{P}-{\bar{W}}_{ep}$.

**Figure 11:**
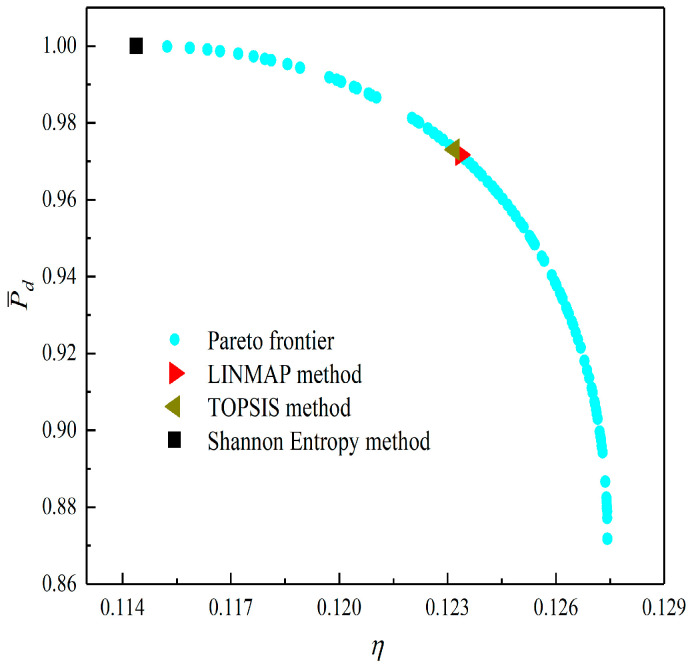
Two-objective optimization on $\eta -{\bar{P}}_{d}$.

**Figure 12:**
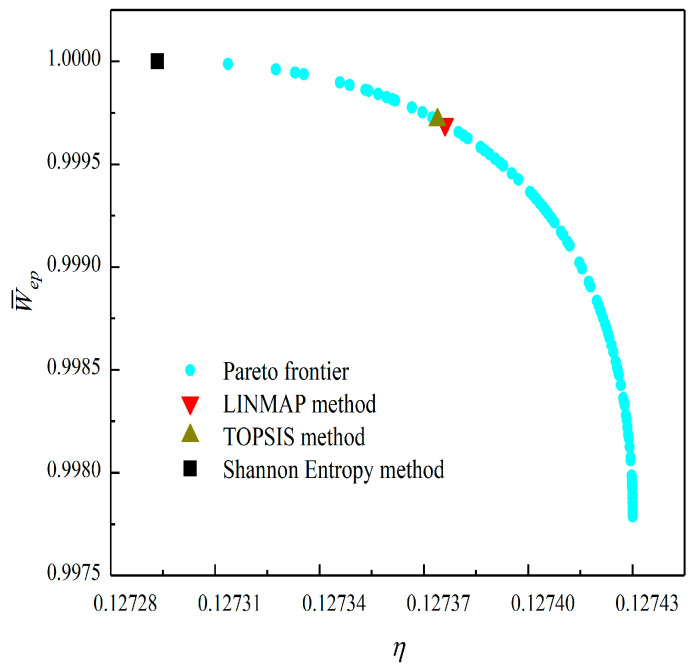
Two-objective optimization on $\eta -{\bar{W}}_{ep}$.

**Figure 13:**
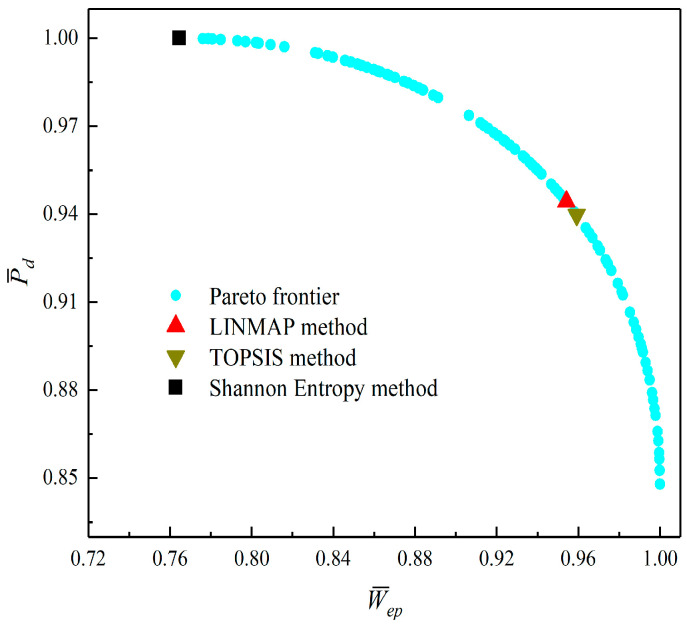
Two-objective optimization on ${\bar{P}}_{d}-{\bar{W}}_{ep}$.

Figure 8, Figure 9, Figure 10, Figure 11, Figure 12 and Figure 13 show the Pareto optimal frontiers obtained by taking two objectives ($\bar{P}-\eta$, $\bar{P}-{\bar{P}}_{d}$, $\bar{P}-{\bar{W}}_{ep}$, $\eta -{\bar{P}}_{d}$, $\eta -{\bar{W}}_{ep}$, ${\bar{P}}_{d}-{\bar{W}}_{ep}$) as the optimization goals. It can be seen from the curves that as $\bar{P}$ increases, η, ${\bar{P}}_{d}$, and ${\bar{W}}_{ep}$ will decrease; as η increases, ${\bar{P}}_{d}$ and ${\bar{W}}_{ep}$ will decrease; and as ${\bar{W}}_{ep}$ increases, ${\bar{P}}_{d}$ will decrease. Comparing the results of the two-objective optimizations in Table 1, it can be found that when the MOO of ${\bar{P}}_{d}$ and ${\bar{W}}_{ep}$ is performed, the deviation index under the TOPSIS method is the minimum.

**Figure 14:**
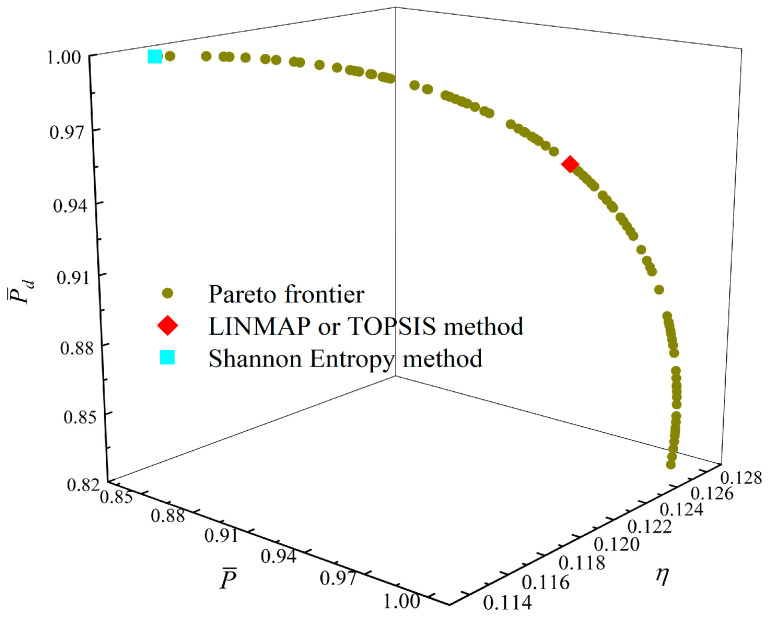
Three-objective optimization on $\bar{P}-\eta -{\bar{P}}_{d}$.

**Figure 15:**
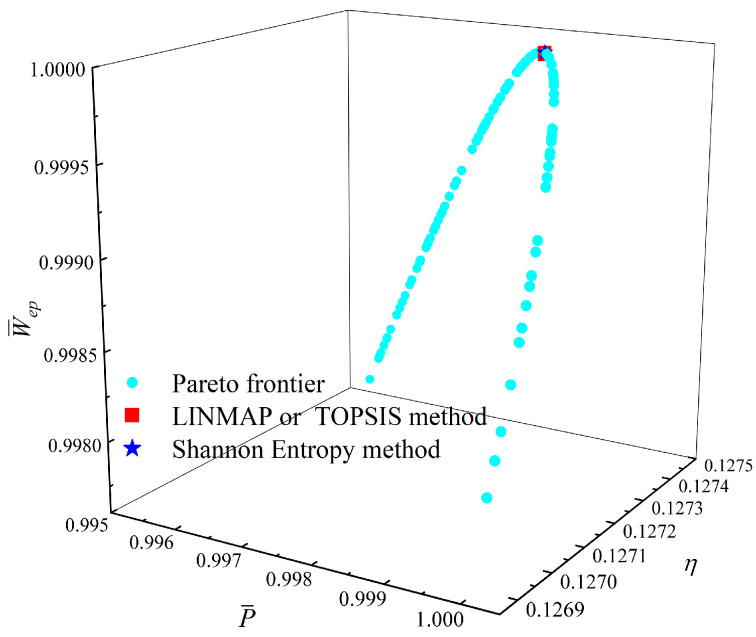
Three-objective optimization on $\bar{P}-\eta -{\bar{W}}_{ep}$.

**Figure 16:**
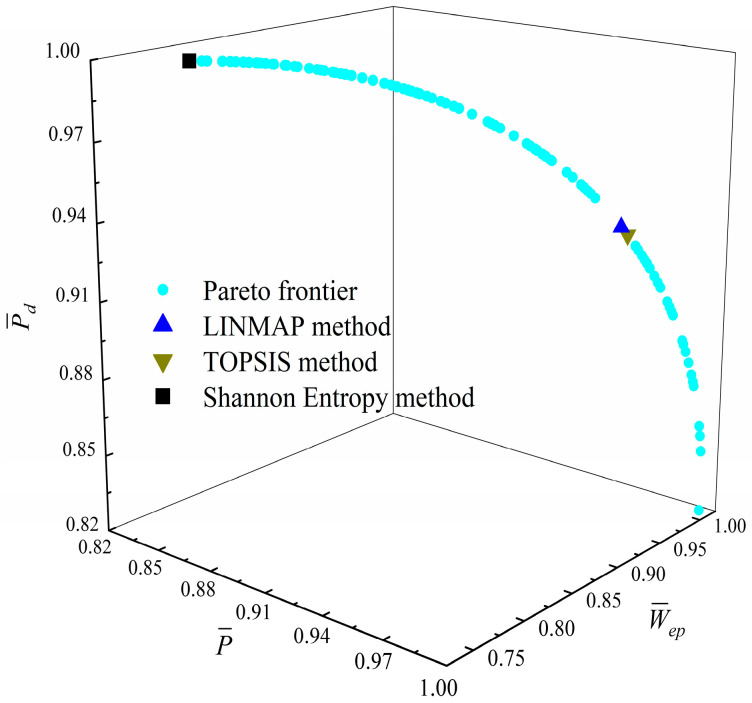
Three-objective optimization on $\bar{P}-{\bar{P}}_{d}-{\bar{W}}_{ep}$.

**Figure 17:**
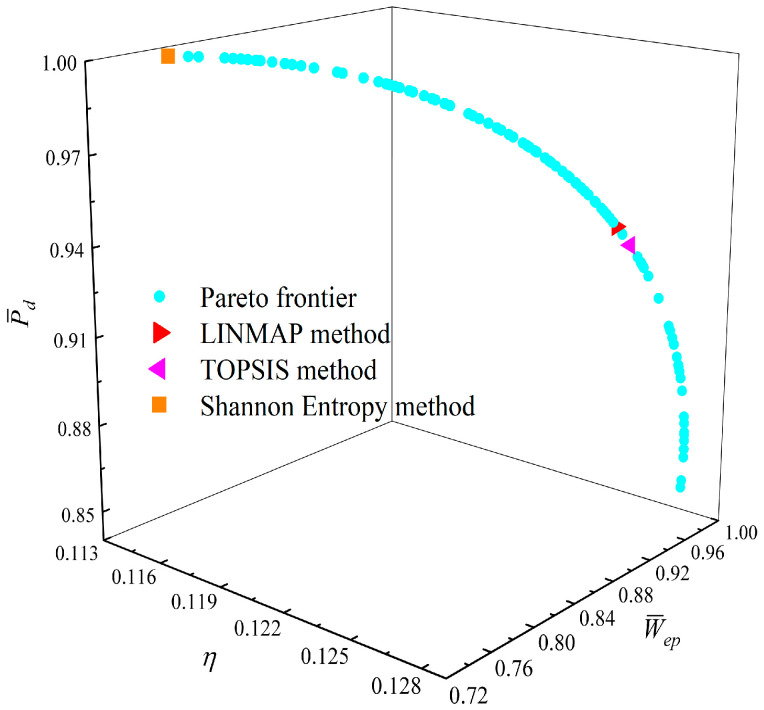
Three-objective optimization on $\eta -{\bar{P}}_{d}-{\bar{W}}_{ep}$.

Figure 14, Figure 15, Figure 16 and Figure 17 show the Pareto optimal frontiers obtained by taking three objectives ($\bar{P}-\eta -{\bar{P}}_{d}$, $\bar{P}-\eta -{\bar{W}}_{ep}$, $\bar{P}-{\bar{P}}_{d}-{\bar{W}}_{ep}$, $\eta -{\bar{P}}_{d}-{\bar{W}}_{ep}$) as the optimization goals. It can be seen from the curves that as $\bar{P}$ and η increase, ${\bar{P}}_{d}$ will decrease; as $\bar{P}$ increases and η decreases, ${\bar{W}}_{ep}$ will first increase and then decrease; as $\bar{P}$ and ${\bar{W}}_{ep}$ increase, ${\bar{P}}_{d}$ will decrease; and as η and ${\bar{W}}_{ep}$ increase, ${\bar{P}}_{d}$ will decrease. Comparing the results of the three-objective optimizations in Table 1, it can be found that when the MOO of $\bar{P}$, ${\bar{P}}_{d}$, and ${\bar{W}}_{ep}$ is performed, the deviation index under the TOPSIS method is the minimum.

**Figure 18:**
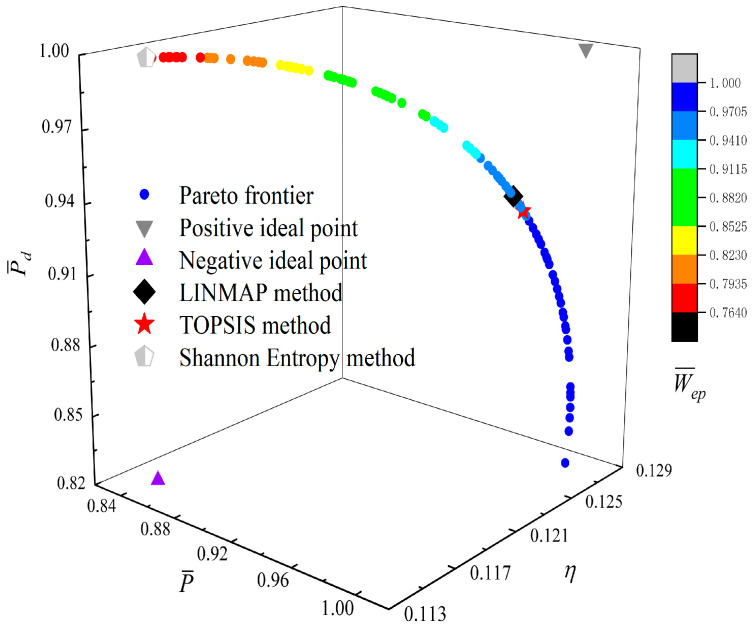
Pareto frontier corresponding to four-objective optimization.

Figure 18 shows the Pareto optimal frontier obtained by taking $\bar{P}$, ${\bar{P}}_{d}$, η, and ${\bar{W}}_{ep}$ of the RC as the optimization goals. In this figure, the positive ideal point in the figure means that ${\bar{P}}_{d}$, $\bar{P}$, η, and ${\bar{W}}_{ep}$ can all reach the maximum, and the negative ideal point means that ${\bar{P}}_{d}$, $\bar{P}$, η, and ${\bar{W}}_{ep}$ can all reach the minimum. It can be seen from the figure that both the positive and negative ideal points are all located outside the Pareto frontier, so there is no optimal γ which makes ${\bar{P}}_{d}$, $\bar{P}$, η, and ${\bar{W}}_{ep}$ all reach the maximum or minimum. Additionally, for the four-objective optimization, the optimization result obtained by using the TOPSIS method has a larger $\bar{P}$, η, and ${\bar{W}}_{ep}$, while the Shannon Entropy decision method has a larger ${\bar{P}}_{d}$; in other words, the corresponding heat engine size is smaller. Comparing the results of the four-objective optimizations in Table 1, it can be found that the deviation index under the TOPSIS method is the minimum.

**Figure 19:**
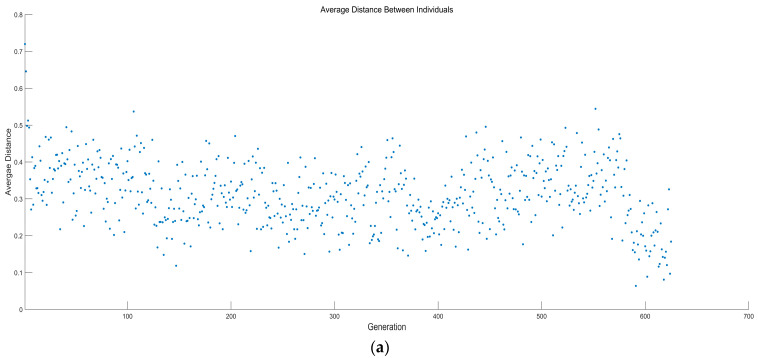

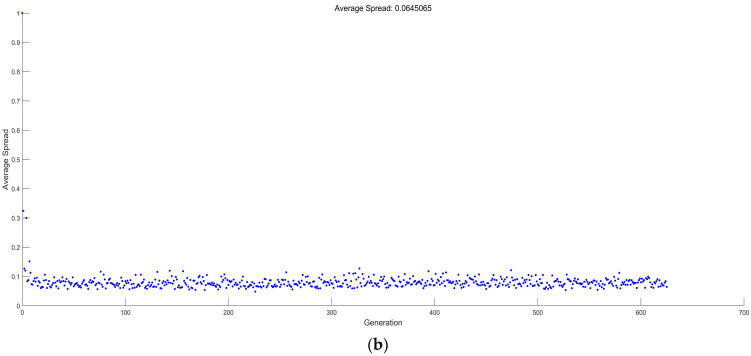
Average distance and average spread versus number of generations (four-objective optimization). (**a**) Average distance versus number of generations (four-objective optimization). (**b**) Average spread versus number of generations (four-objective optimization).

**Figure 20:**
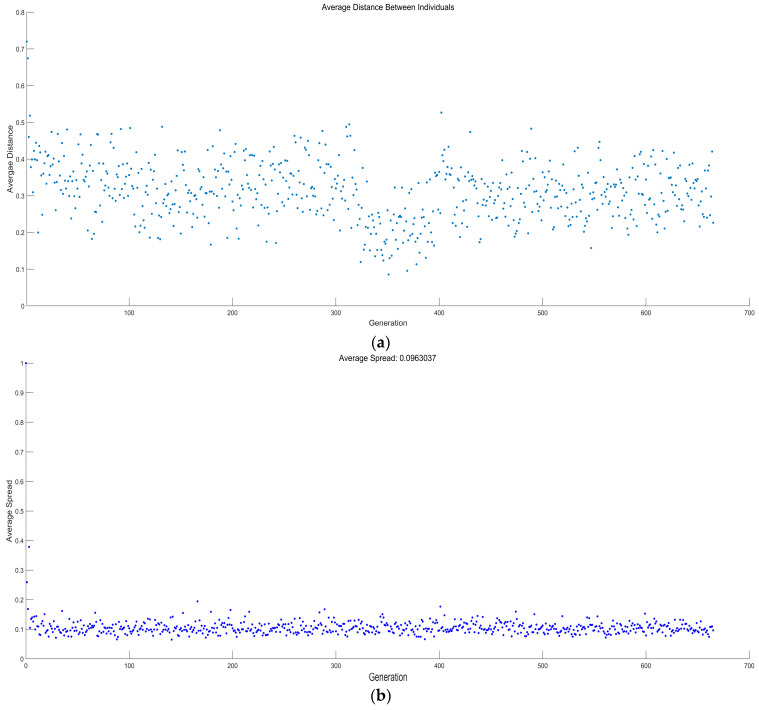
Average distance and average spread versus number of generations (three-objective optimization on $\bar{P}$, ${\bar{P}}_{d}$, and ${\bar{W}}_{ep}$). (**a**) Average distance versus number of generations (three-objective optimization on $\bar{P}$, ${\bar{P}}_{d}$, and ${\bar{W}}_{ep}$). (**b**) Average spread versus number of generations (three-objective optimization on $\bar{P}$, ${\bar{P}}_{d}$, and ${\bar{W}}_{ep}$).

Figure 19 and Figure 20 show the average distance and average spread versus generations for two different multi-objective optimizations. It can be seen from the figures that the genetic algorithm stops when convergence is attained, and it can be observed that this occurs at 626 and 666 generations for the four-objective optimization and three-objective optimization on $\bar{P}$, ${\bar{P}}_{d}$, and ${\bar{W}}_{ep}$.

## 5. Conclusions

Based on the established irreversible RC model, the analytical expressions of cycle PD and effective power are obtained, and the performance comparisons of the cycle under the conditions of the maximum PD, maximum effective power, and maximum POW are compared. The four objectives of the dimensionless POW, TEF, dimensionless PD, and dimensionless effective power are optimized, and the optimal solutions of four-objective, three-objective, and two-objective optimizations are compared. The results show that:

(1) Compared with the maximum POW condition, although part of the TEF is sacrificed when the heat engine works under the maximum PD condition, the heat engine’s size reduces greatly, which has certain guidance for the actual design of the heat engine.
(2) Compared with the maximum POW condition, the TEF is higher when the cycle works under the maximum effective power condition, the TEF can increase with sacrificing part of the POW under the maximum effective power condition, and the effective power reflects the compromise between the POW and TEF.
(3) Comparing the results of four-objective, three-objective, two-objective, and one-objective optimizations, when MOO is performed on dimensionless POW, dimensionless PD, and dimensionless effective power, the deviation index obtained from the TOPSIS decision-making method is the smallest value. At this time, the deviation index is 0.2348, and the optimal compression ratio is 2.1077, which means that the result is the best, and the multi-objective optimization solution is better than the single objective optimal solutions.

## Figures and Tables

**Table 1:** The optimal solutions obtained by single, double, triple, and quadruple objective optimizations.

Optimization Methods	Solutions	Optimization Variable	Optimization Objectives				Deviation Index
		γ	$\bar{P}$	η	${\bar{P}}_{d}$	${\bar{W}}_{ep}$	D
Quadruple objective optimization ($\bar{P}$, η, ${\bar{P}}_{d}$, and ${\bar{W}}_{ep}$)	LINMAP	2.0893	0.9699	0.1260	0.9612	0.9379	0.2355
	TOPSIS	2.1115	0.9738	0.1263	0.9672	0.9317	0.2350
	Shannon Entropy	1.7170	0.8499	0.1144	0.7645	1	0.6142
Triple objective optimization ($\bar{P}$,η, and ${\bar{P}}_{d}$)	LINMAP	2.0357	0.9594	0.1252	0.9443	0.9521	0.2543
	TOPSIS	2.0357	0.9594	0.1252	0.9443	0.9521	0.2543
	Shannon Entropy	1.7170	0.8499	0.1144	0.7645	1	0.6142
Triple objective optimization ($\bar{P}$, η, and ${\bar{W}}_{ep}$)	LINMAP	2.3800	0.9990	0.1273	0.9999	0.8480	0.3519
	TOPSIS	2.3800	0.9990	0.1273	0.9999	0.8480	0.3519
	Shannon Entropy	2.3802	0.9990	0.1273	1	0.8479	0.3520
Triple objective optimization ($\bar{P}$, ${\bar{P}}_{d}$, and ${\bar{W}}_{ep}$)	LINMAP	2.0965	0.97124	0.1261	0.9632	0.9359	0.2349
	TOPSIS	2.1077	0.9732	0.1263	0.9662	0.9328	0.2348
	Shannon Entropy	1.7170	0.8499	0.1144	0.7645	1	0.6142
Triple objective optimization (η, ${\bar{P}}_{d}$, and ${\bar{W}}_{ep}$)	LINMAP	2.0725	0.9668	0.1258	0.9562	0.9425	0.2385
	TOPSIS	2.0963	0.9712	0.12610	0.9631	0.9360	0.2349
	Shannon Entropy	1.7170	0.8499	0.1144	0.7645	1	0.6142
Double objective optimization ($\bar{P}$ and η)	LINMAP	2.3795	0.9990	0.1273	0.9999	0.8481	0.3516
	TOPSIS	2.3795	0.9990	0.1273	0.9999	0.8481	0.3516
	Shannon Entropy	2.3074	0.9957	0.1274	0.9978	0.8718	0.3174
Double objective optimization ($\bar{P}$ and ${\bar{P}}_{d}$)	LINMAP	2.0107	0.9538	0.1247	0.9352	0.9583	0.2719
	TOPSIS	2.0034	0.9521	0.1245	0.9324	0.9600	0.2781
	Shannon Entropy	1.7170	0.8499	0.1144	0.7645	1	0.6142
Double objective optimization ($\bar{P}$ and ${\bar{W}}_{ep}$)	LINMAP	2.4074	0.9996	0.1272	0.9997	0.8389	0.3657
	TOPSIS	2.4061	1	0.1268	0.9975	0.8209	0.3923
	Shannon Entropy	2.3802	0.9990	0.1273	1	0.8479	0.3520
Double objective optimization (*η* and ${\bar{P}}_{d}$)	LINMAP	1.9519	0.9388	0.1234	0.9107	0.9717	0.3313
	TOPSIS	1.9453	0.9370	0.1232	0.9077	0.9731	0.3391
	Shannon Entropy	1.7170	0.8499	0.1144	0.7645	1	0.6142
Double objective optimization (η and ${\bar{W}}_{ep}$)	LINMAP	2.3529	0.9980	0.1274	0.9997	0.8569	0.3381
	TOPSIS	2.3538	0.9981	0.1274	0.9997	0.8566	0.3385
	Shannon Entropy	2.3802	0.9990	0.1273	1	0.8479	0.3520
Double objective optimization (${\bar{P}}_{d}$ and ${\bar{W}}_{ep}$)	LINMAP	2.0657	0.9655	0.1257	0.9542	0.9443	0.2405
	TOPSIS	2.0825	0.9687	0.1259	0.9592	0.9397	0.2364
	Shannon Entropy	1.7170	0.8499	0.1144	0.7645	1	0.6142
Maximum $\bar{P}$	——	2.4061	1	0.1268	0.9975	0.8209	0.3923
Maximum η	——	2.3529	0.9980	0.1274	0.9997	0.8569	0.3381
Maximum ${\bar{P}}_{d}$	——	2.3802	0.9990	0.1273	1	0.8479	0.3520
Maximum ${\bar{W}}_{ep}$	——	1.7170	0.8499	0.1144	0.7645	1	0.6142
Positive ideal point	——	——	1	0.1274	1	1	——
Negative ideal point	——	——	0.8499	0.1144	0.7645	0.8244	——

## Data Availability

Not applicable.

## Nomenclature

B	Heat transfer loss coefficient (W/K)
$C_{p}$	Specific heat at constant pressure (J/(mol⋅K))
$C_{v}$	Specific heat at constant volume (J/(mol⋅K))
P	Power output (W)
*P_d_*	Power density (${W/m}^{3}$)
$\dot{Q}$	Heat transfer rate (W)
T	Temperature (K)
$W_{ep}$	Efficient power (W)
Greek symbols	
γ	Compression ratio (-)
η	Thermal efficiency (-)
μ	Friction coefficient (kg/s)
τ	Temperature ratio (-)
Subscripts	
in	Input
leak	Heat leak
out	Output
$\bar{P}$	Max dimensionless power output condition
${\bar{P}}_{d}$	Max dimensionless power density condition
${\bar{W}}_{ep}$	Max dimensionless effective power condition
μ	Influence of friction loss
1−4	Cycle state points
Superscripts	
−	Dimensionless

## Abbreviations

MOO	Multi-objective optimization
PD	Power density
POW	Power output
RC	Rectangular cycle
SH	Specific heat
TEF	Thermal efficiency
TR	Temperature ratio
WF	Working fluid
